# Intravenous esketamine as an adjuvant for sedation/analgesia outside the operating room: a systematic review and meta-analysis

**DOI:** 10.3389/fphar.2024.1287761

**Published:** 2024-07-03

**Authors:** Ziheng Kan, Weixiang Min, Yuee Dai, Peng Zhang

**Affiliations:** ^1^ School of Medicine, University of Electronic Science and Technology of China, Chengdu, China; ^2^ Department of Anesthesiology, Sichuan Provincial People’s Hospital, School of Medicine, University of Electronic Science and Technology of China, Chengdu, China

**Keywords:** esketamine, sedation, analgesia, outside of operating room, propofol

## Abstract

**Background:**

This study was conducted to evaluate the safety and efficacy of intravenous esketamine as an adjuvant for sedation or analgesia outside the operating room in adults and children.

**Method:**

PubMed, Embase, Cochrane Central Register of Controlled Trials (CENTRAL), Web of Science, and Scopus were searched for potential randomized controlled studies randomized controlled trials comparing drug combinations of esketamine to any other single or combination drug regimens for sedation or analgesia outside the operating room.

**Results:**

Twenty-five studies with a total of 3,455 participants were included in this review. The pooled results of adults showed that compared with drug regimens of the control group, intravenous esketamine combinations were significantly associated with decreased risk of oxygen desaturation (RR = 0.49, 95% CI = [0.34, 0.70]); hypotension (RR = 0.38, 95% CI = [0.31, 0.46]); bradycardia (RR = 0.23, 95% CI = [0.12, 0.43]); injection pain (RR = 0.37, 95% CI = [0.25, 0.53]); body movement (RR = 0.60, 95% CI = [0.41, 0.88]); and propofol consumption (SMD = −1.38, 95% CI = [−2.64, −0.11]), but an increased risk of psychiatric symptoms (RR = 3.10, 95% CI = [2.11, 4.54]) (RR = relative risk; CI = confidence intervals; SMD = standardized mean difference). Subgroup analysis showed that only the combination of esketamine and propofol significantly reduced the above incidence of respiratory and cardiovascular adverse events in adults. In addition, the pooled results of children showed that compared with drug regimens of the control group, esketamine and propofol co-administration significantly reduced the risk of hypotension (RR = 0.59, 95% CI = [0.37, 0.95]) but increased the risk of visual disturbance (RR = 6.62, 95% CI = [2.18, 20.13]) and dizziness (RR = 1.99, 95% CI = [1.17, 3,37]). Subgroup analysis indicated that esketamine>0.5 mg/kg significantly reduced the incidence of hypotension, but increased the risk of dizziness in children.

**Conclusion:**

Intravenous use of esketamine, particularly in combination with propofol, may improve the safety and efficacy of sedation and analgesia outside the operating room, although the potential for psychiatric side effects warrants attention. Future research is recommended to investigate the role of esketamine with agents other than propofol.

## 1 Introduction

The need for sedation or analgesia outside the operating room is increasing because of improvements in medical technology (Pino, 2007). Various drugs such as propofol, benzodiazepines, opioids, ketamine, and dexmedetomidine, either alone or in combination, have been used (Jo and Kwak, 2019). Combination drugs help to optimize the desired effects while countering each other’s side effects (Hinkelbein et al., 2020). The common administration of opioid-propofol combination and other drug combinations have been shown to be more effective than single agents for sedation or analgesia (Boriosi et al., 2017; Hayes et al., 2021; Rathi et al., 2022; De Vries et al., 2023).

Esketamine, an antagonist of the N-methyl-D-aspartic acid (NMDA) receptor, is an s-enantiomer of ketamine. It produces approximately twice higher sedative activity but induces fewer side effects than ketamine (Peltoniemi et al., 2016). Esketamine and ketamine can deliver analgosedation action, but some drawbacks such as the induction of frequent vomiting and clustered psychiatric symptoms limit their single administration (Nakao et al., 2003). Low-dose esketamine as an adjuvant has been shown to have several benefits for sedation or anesthesia, including stabilizing hemodynamics and respiratory function, decreasing propofol requirements, or reducing postoperative pain sensitivity (Huang et al., 2023; Ren et al., 2023).

In this study, we conducted an updated systematic review and meta-analysis to include all drug combinations of intravenous esketamine for sedation or analgesia outside the operating room. We also aimed to compare the safety and efficacy of combination regimens of esketamine with other drug regimens of the control group in adults and children undergoing various types of surgical procedures outside the operating room.

## 2 Materials and methods

### 2.1 Literature search and selection criteria

This review was based on the Preferred Reporting Items for Systematic Reviews and Meta-Analyses (PRISMA) guidelines (Moher et al., 2010). The PubMed, Embase, Cochrane Central Register of Controlled Trials (CENTRAL), Web of Science, and Scopus databases were searched for potential randomized controlled trials (RCTs) that assessed the effect of esketamine on sedation or analgesia in adults and children undergoing surgical procedures outside the operating room. The date parameters for the search were set from database inception to 19 Marc^h^ 2024. The search strategy was specific to each database and is available in Supplementary Material S1. Additional studies in the reference lists of selected articles were also screened for possible eligibility. Clinicaltrials.gov was also searched for possible ongoing studies. In our study, the PICOS criteria are as follows: Population (P): patients requiring sedation or analgesia in clinical settings outside the operating room; Intervention (I): intravenous administration of esketamine in combination with other sedative or analgesic agents; Comparator (C): other drug regimens without the adjunctive use of esketamine; Outcomes (O): the primary outcomes of interest were the safety of using esketamine for sedation or analgesia outside the operating room, particularly examining respiratory and cardiovascular adverse events; Study design (S): our study is based on RCTs.

The inclusion criteria were as follows: full-text available RCTs, intravenous esketamine in combination with other anesthetics, and studies with participants who had undergone sedation or analgesia outside the operating room. The exclusion criteria were as follows: failure to provide sufficient information or data, sedation performed in the ICU or emergency room, or studies with participants under general anesthesia with laryngeal insertion or tracheal intubation.

### 2.2 Data collection

Two independent authors extracted data from the included studies based on a previously designed data extraction table. The following information was extracted: author, publication year, number of participants, age, intervention measures, type of surgery, and outcomes. The primary outcomes were respiratory and cardiovascular adverse events. The secondary outcomes were injection pain, inadequate sedation parameters (intraoperative cough and body movement), nausea and/or vomiting, propofol consumption, psychiatric symptoms, visual disturbance, dizziness, emergence delirium, awakening time, recovery time, and orientation recovery time. Where data were presented as values other than the mean and standard deviations, we attempted to contact the author to obtain raw data. If this was not possible, data were excluded from the meta-analysis. In the case of studies with more than one treatment group (including different doses of esketamine), we combined different doses as the esketamine≤0.5 mg/kg subgroup or esketamine>0.5 mg/kg subgroup (Higgins and Deeks, 2011). The risk of bias of the included studies was also independently assessed by two reviewers using the Cochrane risk of bias tool. Five parameters, namely, randomization sequence generation, allocation concealment, blinding, incomplete outcome data, and selective reporting, from each included study were evaluated as low, unclear, or high risk of bias. The database searching, literature screening, data collection and risk of bias assessment were conducted independently by two authors, ZK and WM. Any discrepancies were resolved by discussion with the third author, PZ.

### 2.3 Statistical analysis

The relative risk (RR) with 95% confidence intervals (CI) was calculated for dichotomous data. The standardized mean difference (SMD) with 95% CI was used to express continuous variables owing to differences in measurement scales and methods of drug administration across included studies. Differences were considered statistically significant if *p* < 0.05, 95%CI of RR excluded 1, or 95% CI excluded 0 for the SMD. The I^2^ statistic was used to assess heterogeneity. If heterogeneity was significant (I^2^>50%), a random effects model was used; otherwise, a fixed effects model was used. Subgroup analysis was conducted according to the esketamine drug regimens (esketamine combined with propofol or other drugs) and esketamine dose (≤0.5 mg/kg or >0.5 mg/kg). We did not perform subgroup analysis if there were fewer than two trials in each subgroup. Additionally, sensitivity analysis was performed by omitting one study each time for primary outcomes to identify potential sources of heterogeneity. Publication bias was assessed by visual inspection of funnel plots. Statistical analyses were performed using Review Manager (RevMan) version 5.1 (Copenhagen: The Nordic Cochrane Centre, The Cochrane Collaboration, 2011).

## 3 Results

### 3.1 Study search and characteristics

**FIGURE 1:**
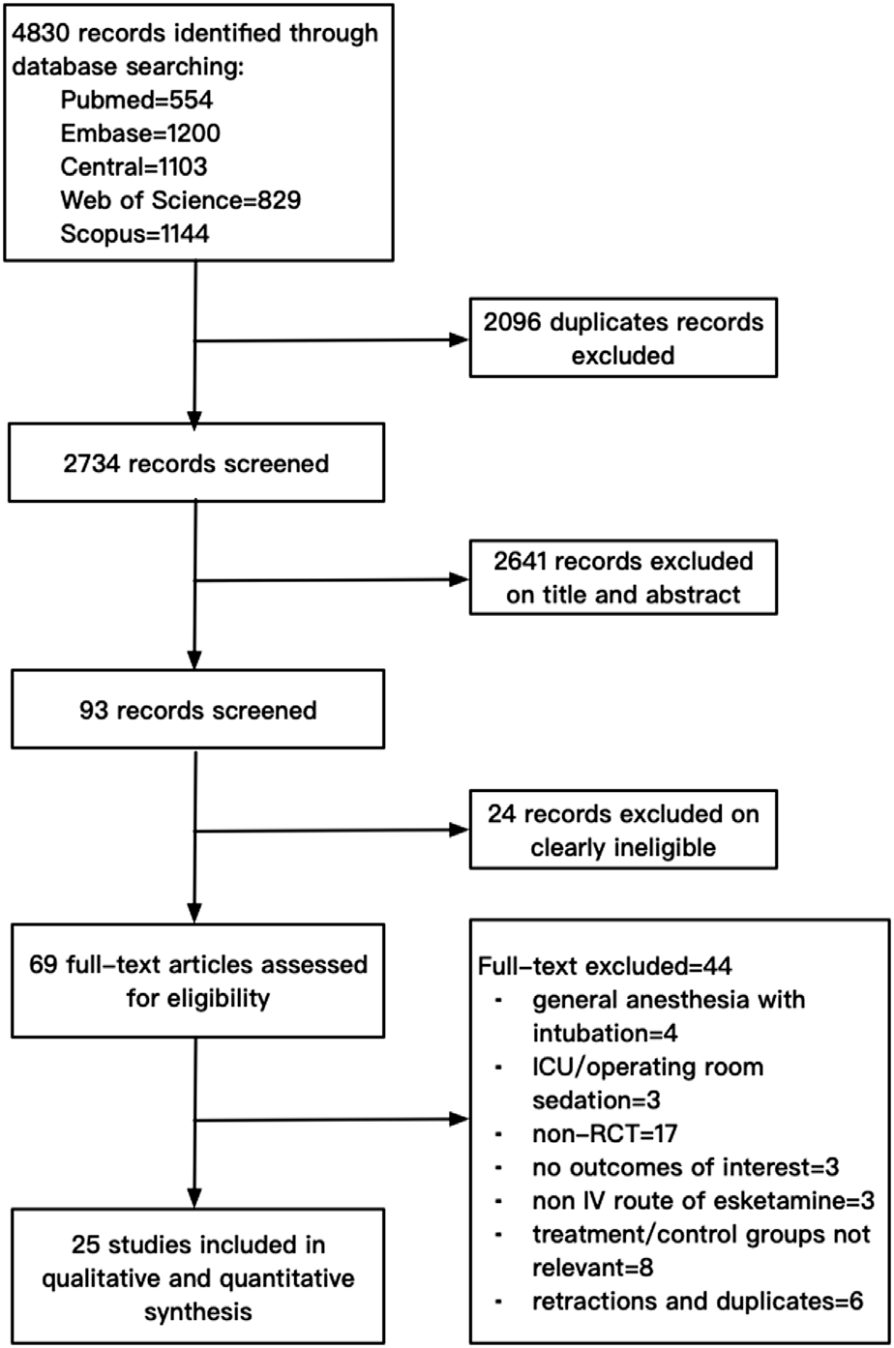
A flowchart of the literature search for the included studies.

The electronic search yielded a total of 4,830 citations. After excluding 2096 duplicates, 2,734 studies were screened. After screening the titles and abstracts and assessing the full texts, 25 studies with a total of 3,455 participants were finally included in this review. A flowchart of the literature search for the included studies is shown in Figure 1. The data of 44 studies excluded by full-text screening are available in Supplementary Material S2.

Six studies involved 698 pediatric participants (age range: 2 days to 12 years) (Pees et al., 2003; Wang et al., 2022; Xu (b) et al., 2022; Zheng (b) et al., 2023; Zheng et al., 2022; Zhong et al., 2023), and the 19 remaining studies involved 2,757 adult participants (18–89 years) (Chen et al., 2022; Chen et al., 2023; Cui et al., 2023; Eberl et al., 2020; Feng (a) et al., 2022; Feng (b) et al., 2022; Lin et al., 2023; Liu et al., 2023; Lu et al., 2022; Ma et al., 2024; Nie et al., 2023; Si et al., 2024; Song et al., 2023; Wang et al., 2019; Xin et al., 2024; Xu (a) et al., 2022; Yang et al., 2022; Zhan et al., 2022; Zheng (a) et al., 2023). Fifteen studies carried out diagnostic and therapeutic gastrointestinal endoscopy (Chen et al., 2023; Eberl et al., 2020; Feng (a) et al., 2022; Liu et al., 2023; Lu et al., 2022; Ma et al., 2024; Song et al., 2023; Wang et al., 2022; Wang et al., 2019; Xu (a) et al., 2022; Yang et al., 2022; Zhan et al., 2022; Zheng (a) et al., 2023; Zheng (b) et al., 2023; Zheng et al., 2022); four conducted fiberoptic bronchoscopy (Cui et al., 2023; Feng (b) et al., 2022; Nie et al., 2023; Zhong et al., 2023); and six studies involved abortions (Chen et al., 2022), cervical conization (Si et al., 2024), MRI (Xu (b) et al., 2022), cardiac catheterization (Pees et al., 2003), dental extraction (Xin et al., 2024), and percutaneous radiofrequency ablation of lung tumor (Lin et al., 2023), respectively. Twenty-two studies used a combination of esketamine and propofol (Chen et al., 2022; Chen et al., 2023; Cui et al., 2023; Eberl et al., 2020; Feng (a) et al., 2022; Feng (b) et al., 2022; Liu et al., 2023; Ma et al., 2024; Nie et al., 2023; Si et al., 2024; Song et al., 2023; Wang et al., 2022; Wang et al., 2019; Xin et al., 2024; Xu (a) et al., 2022; Xu (b) et al., 2022; Yang et al., 2022; Zhan et al., 2022; Zheng (a) et al., 2023; Zheng (b) et al., 2023; Zheng et al., 2022; Zhong et al., 2023), two studies used esketamine plus benzodiazepines (Pees et al., 2003; Lu et al., 2022), and one study used esketamine combined with dexmedetomidine (Lin et al., 2023). Eighteen studies involved one esketamine group with doses ranging from 0.15 to 1.0 μg/kg (Cui et al., 2023; Eberl et al., 2020; Feng (b) et al., 2022; Lin et al., 2023; Liu et al., 2023; Lu et al., 2022; Ma et al., 2024; Nie et al., 2023; Pees et al., 2003; Si et al., 2024; Song et al., 2023; Wang et al., 2019; Xin et al., 2024; Xu (a) et al., 2022; Xu (b) et al., 2022; Zheng (a) et al., 2023; Zheng (b) et al., 2023; Zhong et al., 2023), and seven studies involved more than one esketamine group with doses ranging from 0.05 to 1.0 μg/kg (Chen et al., 2022; Chen et al., 2023; Feng (a) et al., 2022; Wang et al., 2022; Yang et al., 2022; Zhan et al., 2022; Zheng et al., 2022). With respect to drug regimens in the control groups, 10 studies used propofol alone (Cui et al., 2023; Feng (a) et al., 2022; Liu et al., 2023; Nie et al., 2023; Wang et al., 2022; Yang et al., 2022; Zhan et al., 2022; Zheng (a) et al., 2023; Zheng (b) et al., 2023; Zheng et al., 2022); 13 studies used propofol in combination with opioids (Chen et al., 2022; Chen et al., 2023; Eberl et al., 2020; Feng (b) et al., 2022; Lu et al., 2022; Ma et al., 2024; Si et al., 2024; Song et al., 2023; Xu (a) et al., 2022; Zhong et al., 2023), ketamine (Wang et al., 2019), or dexmedetomidine (Xin et al., 2024; Xu (b) et al., 2022); one study used ketamine and midazolam (Pees et al., 2003); and one study used sufentanil in combination with dexmedetomidine (Lin et al., 2023). The characteristics of the included studies are shown in Table 1.

**TABLE 1:** The characteristics of the included studies.

No.	Study	Procedure	No.	Age	Treatment group	Control group	Outcomes
1	Zhan et al. (2022)	Gastrointestinal endoscopy	260	18–60y	esketamine (0.05, 0.1, 0.2 ug/kg) + propofol 1.5 mg/kg	propofol 1.5 mg/kg	propofol consumption, procedure time, induction time, SBP, DBP, HR, SpO_2_, awakening delay, orientation recovery time, adverse events, psychotomimetic effects, MMSE
2	Zheng et al. (2022)	diagnostic upper gastrointestinal endoscopy	92	3–12y	esketamine (0.25, 0.5, 1.0 ug/kg) + propofol	propofol	ED50 of propofol, propofol doses, awakening time, examination time, MAP, HR, adverse events, physician and patient satisfaction
3	Feng (a) 2022	Gastrointestinal endoscopy	100	18–65y	esketamine (0.15, 0.25, 0.5 ug/kg) + propofol TCI	propofol TCI	EC50 of propofol, propofol doses, procedure time, awakening time, MAP, HR, adverse effects
4	Yang et al. (2022)	gastrointestinal endoscopy	90	65–89y	esketamine (0.25, 0.5 ug/kg) + propofol	propofol TCI	EC50 of propofol, change of MAP and HR, recovery time, procedure time, patient and gastroenterologist satisfaction, adverse effects, psychotomimetic effects
5	Wang et al. (2022)	gastro-duodenoscopy	120	6–12y	esketamine (0.3, 0.5, 0.7 ug/kg) + propofol 3 mg/kg	propofol 3 mg/kg	procedure duration, number of cases with smooth placement of first endoscope insertion, times of additional propofol, propofol dose, recovery time, PACU stay, endoscopist satisfaction rate, MAP, HR, BIS, adverse events
6	Lu et al. (2022)	gastroenteroscopy	106	20–75y	esketamine 1.0 ug/kg + remimazolam 0.3–0.4 mg/kg	sufentanil 0.1 ug/kg + propofol	induction time, awakening time, orientation recovery time, adverse effects
7	Ma et al. (2024)	endoscopic resection in colorectum	166	>18y	propofol TCI 1.5−2.5 mg/mL + esketamine 0.15 mg/kg	propofol TCI 1.5−2.5 mg/mL + fentanyl 1ug/kg	propofol consumption, vasoactive drug dosages, sedation-related times, adverse events, and satisfaction
8	Si et al. (2024)	cervical conization	122	18-60	esketamine 0.15 mg/kg + sufentanil 0.1ug/kg +propofol	sufentanil 0.2ug/kg + propofol	incidence and severity of SRAEs, effectiveness of sedation, awakening time, psychotomimetic side effects, postoperative pain, PONV, and patient and gynaecologist satisfaction
9	Feng (b) 2022	fibronchoscopy	80	>65y	esketamine 0.15 ug/kg + propofol	sufentanil 0.1ug/kg+ propofol	MAP, HR and SpO_2_, propofol dose, examination time, wake-up time, VAS score, adverse reactions
10	Chen et al. (2022)	abortion	178	18–45y	esketamine (0.2, 0.25, 0.3 ug/kg) + propofol	fentanyl 1 mg/kg + propofol 2 mg/kg	incidence of complications, MAP, HR, SpO2, surgery time, induction time, recovery time, dischargeable time, frequency of additional propofol, propofol dose, satisfaction (patient, surgeon, anesthesiologist), postoperative pain
11	Xu (a) 2022	gastroscopy	87	18–64y	esketamine 0.3 ug/kg + propofol	dezocine 0.05 mg/kg+ propofol	total dose of propofol, endoscopy time, recovery time, endoscopist and patient satisfaction, MAP, HR, SpO2, side effects
12	Eberl et al. (2020)	ERCP	162	>18y	esketamine 0.15 ug/kg + propofol (TCI)	alfentanil 2ug/kg + propofol (TCI)	procedure duration, propofol dose, recovery time, patient and endoscopist satisfaction, patient recommendation, endoscopist perception of patient pain, side effects
13	Wang et al. (2019)	gastroscopy	32	32 ± 6.19y; 40 ± 8.91y	esketamine 0.5 ug/kg + propofol	ketamine 1 mg/kg+ propofol 0.6 mg/kg	pharmacokinetics parameters, recovery time, orientation recovery time, adverse reactions
14	Pees et al. (2003)	Cardiac catheterization	100	2 days-11y	esketamine 1.0 ug/kg + midazolam	ketamine 1 mg/kg + midazolam 0.5 mg/kg	dosage of S- and R-ketamine, nausea and vomiting, sleeping time, further sedative medication after awakening to suppress psychotic reactions and/or heavy body movements
15	Xu et al. (2022)	MRI	114	6months-8 y	esketamine 0.15 ug/kg + propofol	dexmedetomidine 0.3ug/kg + propofol 1.5 mg/kg	propofol dose, adverse reactions, time to emergence from sedation, time to discharge from recovery room, HR, SBP, DBP, scanning time, radiologist and parent satisfaction scores, Ramsey sedation score
16	Chen et al. (2023)	endoscopic variceal ligation	100	18–70y	esketamine (0.2, 0.3, 0.4 ug/kg) + propofol 1.5 mg/kg	sufentanil 0.1 μg/kg + propofol 1.5 mg/kg	Adverse events, SBP, DBP, MAP, HR, TV, RR, MV, SpO2, operative time, awake time, propofol dose, number of additional propofol dosages, airway excreta, pain NRS, patient and endoscopic physicians satisfaction
17	Zheng et al. (2022)	gastroscopy	113	18–64y	esketamine 0.25 ug/kg + propofol	propofol	propofol consumption, induction time, awakening times, SBP, DBP, HR, SpO2, duration of procedure, orientation recovery time, adverse events, endoscopist and anesthesiologist satisfaction score
18	Zheng (b) 2023	gastrointestinal endoscopy	200	3–12y	esketamine 0.5 mg/kg + propofol 2 mg/kg	nalbuphine 0.2 mg/kg + propofol 2 mg/kg	success rate of the endoscope insertion, HR, MAP respiratory depression, examination and awakening time, physician and patient satisfaction, awakening period and 24 h after examination complicates, PAED scale
19	Zhong et al. (2023)	flexible fibreoptic bronchoscopy	72	<12y	esketamine 0.3 mg/kg +propofol 2–2.5 mg/kg, esketamine 0.3 mg/kg/h, propofol 4–10 mg/kg/h, remifentanil 0.05–0.3ug/kg/min for continuous infusion	propofol 2–2.5 mg/kg, continuous infusion drugs as same as treatment group	oxygen desaturation, intraoperative hemodynamics, duration of induction, procedure and anesthesia, recovery time, the time to the ward from the recovery room, dose of propofol and remifentanil, number of times propofol was added, PAED scores, cough scores, injection pain, laryngospasm, bronchospasm, PONV, vertigo, hallucination, agitation
20	Nie et al. (2023)	fiberoptic bronchoscopy	84	18–65y	esketamine 0.2 mg/kg +propofol 1.5 mg/kg	remifentanil 0.5ug/kg + propofol 1.5 mg/kg	intraoperative hemodynamics, dose of propofol, number of patients used additional propofol, times of giving additional propofol, adverse events, recovery of consciousness, awaken time, duration of examination, satisfaction of patients and bronchoscopists
21	Cui et al. (2023)	endobronchial ultrasound-guided transbronchial needle aspiration	140	18–89y	esketamine 0.3 mg/kg + propofol TCI, esketamine 0.2 mg/kg/h for sedative maintenance	propofol TCI	MAP, lidocaine dose, times of lidocaine sprays, cough score, propofol dosage, patient satisfaction, endoscopist satisfaction, sedation-related adverse and recovery time
22	Liu et al. (2023)	gastroscopy	80	18–64y	esketamine 0.2 mg/kg +propofol 1 mg/kg	propofol 1 mg/kg	total amount of propofol, incidences of injection pain, involuntary movement, hemodynamic and respiratory adverse events during examination, total examination time, recovery time and postoperative adverse effects
23	Lin et al. (2023)	lung tumor percutaneous radiofrequency ablation	44	37–84y	esketamine 0.2 mg/kg + dexmedetomidine 1 μg/kg	sufentanil 0.1ug/kg + dexmedetomidine 1 μg/kg	MOAAS, physical movement pain scale, vital signs, recovery time, radiologist and patient satisfaction, respiratory depression and PONV
24	Xin et al. (2024)	dental extraction	150	<60y	propofol 0.5 mg/kg+ esketamine 0.25 mg/kg, then propofol 2.5 mg/kg/h for maintenance for both groups	propofol 0.5 mg/kg+ dexmedetomidine 0.5−1.0 mg/kg/h or propofol 0.5 mg/kg alone	vital signs, blood gas analysis, BIS, adverse reactions, recovery time, patient satisfaction, and doctor satisfaction
25	Song 2023	bidirectional endoscopy	663	18–70y	esketamine 0.15 mg/kg + sufentanil 0.1 μg/kg + propofol 0.5 mg/kg	sufentanil 0.1 μg/kg + propofol 0.5 mg/kg	Desaturation, hypotension, propofol requirements, postprocedure pain, fatigue, nausea or vomiting, dizziness or headache, hallucination or nightmare, endoscopist satisfaction, and patient satisfaction

Fourteen studies had an overall low risk of bias, 10 studies had an unclear risk of bias, and one study had a high risk of bias. The sources of bias were primarily because of poor reporting of the randomization process and blinding, specifically a lack of description about allocation concealment. The risk of bias of the included studies is presented in Table 2.

**TABLE 2:** The risk of bias of the included studies.

Study	Random sequence generation	Allocation concealment	Blinding of participants and personnel	Blinding of outcome assessment	Incomplete outcome data	Selective reporting
Zhan et al. (2022)	low	low	low	low	low	low
Xu (a) 2022	low	unclear	low	low	low	low
Lu et al. (2022)	low	unclear	unclear	unclear	low	low
Zheng et al. (2022)	low	low	low	low	low	low
Feng (a) 2022	low	low	low	low	low	low
Wang et al. (2019)	low	low	unclear	unclear	low	low
Yang et al. (2022)	low	unclear	unclear	low	low	low
Wang et al. (2022)	low	low	low	low	low	low
Feng (b) 2022	low	unclear	unclear	unclear	low	low
Eberl et al. (2022)	low	low	low	low	low	low
Xu (b) 2022	low	low	low	low	low	low
Pees et al. (2003)	unclear	unclear	unclear	low	high	unclear
Chen et al. (2022)	low	low	low	low	low	low
Chen et al. (2023)	low	low	low	low	low	low
Zheng (a) 2023	low	low	low	low	low	low
Zheng (b) 2023	low	low	low	low	low	low
Zhong et al. (2023)	low	low	low	low	low	low
Nie et al. (2023)	low	unclear	low	unclear	low	low
Cui et al. (2023)	low	low	low	low	low	low
Liu et al. (2023)	low	low	low	low	low	low
Lin et al. (2023)	low	unclear	low	low	low	low
Xin et al. (2024)	unclear	unclear	unclear	unclear	low	low
Song 2023	low	low	low	low	low	low
Ma et al. (2024)	low	unclear	low	low	low	low
Si et al. (2024)	low	unclear	low	low	low	low

### 3.2 Pooled results of adults

**FIGURE 2:**
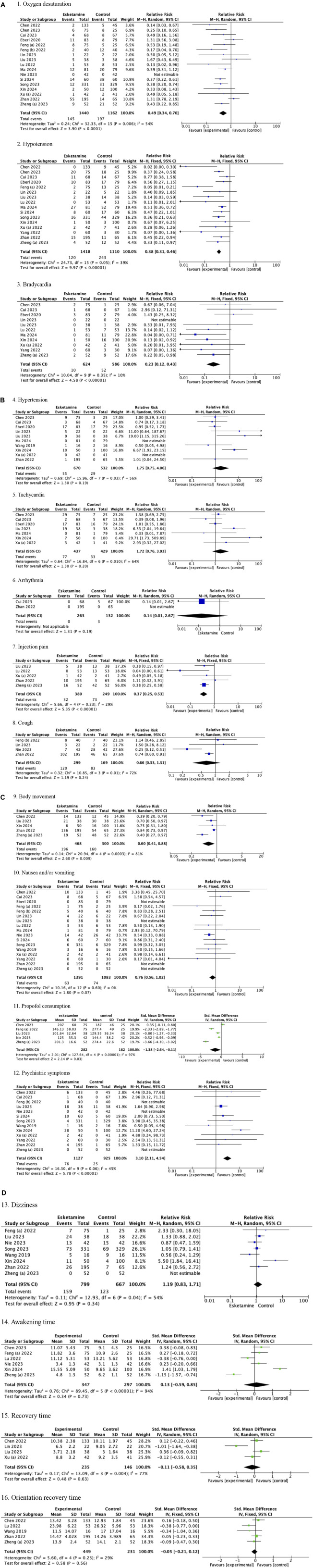
**(A)** Forest plots of adults (1. Oxygen desaturation, 2. Hypotension, and 3. Bradycardia). **(B)** Forest plots of adults (4. Hypertension, 5. Tachycardia, 6. Arrhythmia, 7. Injection pain, and 8. Cough). **(C)** Forest plots of adults (9. Body movement, 10. Nausea and/or vomiting, 11. Propofol consumption, and 12. Psychiatric symptoms). **(D)** Forest plots of adults (13. Dizziness, 14. Awakening time, 15. Recovery time, and 16. Orientation recovery time).

The pooled results in adults showed that compared with drug regimens of the control group, intravenous esketamine drug combinations significantly reduced the risk of oxygen desaturation (RR = 0.49, 95%CI = [0.34, 0.70], *p* < 0.0001); hypotension (RR = 0.38, 95%CI = [0.31, 0.46], *p* < 0.00001); bradycardia (RR = 0.23, 95%CI = [0.12, 0.43], *p* < 0.00001); injection pain (RR = 0.37, 95%CI = [0.25, 0.53], *p* < 0.00001); body movement (RR = 0.60, 95%CI = [0.41, 0.88], *p* = 0.009); and propofol consumption (SMD = −1.38, 95%CI = [−2.64, −0.11], *p* = 0.03) but increased the risk of psychiatric symptoms (RR = 3.10, 95%CI = [2.11, 4.54], *p* < 0.00001). No significant differences were found in hypertension (RR = 1.75, 95%CI = [0.75, 4.06], *p* = 0.19); tachycardia (RR = 1.72, 95%CI = [0.76, 3.93], *p* = 0.20); arrhythmia (RR = 0.14, 95%CI = [0.01, 2.67], *p* = 0.19); cough (RR = 0.66, 95%CI = [0.33, 1.31], *p* = 0.24); nausea and/or vomiting (RR = 0.76, 95%CI = [0.56, 1.02], *p* = 0.07); dizziness (RR = 1.19, 95%CI = [0.83, 1.71], *p* = 0.34); awakening time (SMD = 0.13, 95%CI = [−0.59, 0.85], *p* = 0.73); recovery time (SMD = −0.11, 95%CI = [−0.58, 0.35], *p* = 0.63); or orientation recovery time (SMD = −0.05, 95%CI = [−0.21, 0.12], *p* = 0.56) compared with drug regimens of the control group (Figures 2A–D; Table 3).

**TABLE 3:** Results of meta-analysis in adults and children.

Outcome indicators	Studies	No.	RR or SMD [95%]	*p*-value	I^2^
Adults					
Oxygen desaturation	17 Chen et al. (2022); Chen et al. (2023); Cui et al. (2023); Eberl et al. (2020); Feng et al. (2022); Lin et al. (2023); Liu et al. (2023); Lu et al. (2022); Ma et al. (2024); Nie et al. (2023); Si et al. (2024); Song et al. (2023); Xin et al. (2024); Xu et al. (2022); Zhan et al. (2022); Zheng et al. (2023)	2,602	0.49 [0.34, 0.70 ]	*p* < 0.0001	54%
Hypotension	16 Chen et al. (2022); Chen et al. (2023); Cui et al. (2023); Eberl et al. (2020); Feng et al. (2022); Lin et al. (2023); Liu et al. (2023); Lu et al. (2022); Ma et al. (2024); Si et al. (2024); Song et al. (2023); Xin et al. (2024); Xu et al. (2022); Yang et al. (2022); Zhan et al. (2022); Zheng et al. (2023)	2,528	0.38 [0.31, 0.46]	*p* < 0.00001	39%
Bradycardia	11 Chen et al. (2023); Cui et al. (2023); Eberl et al. (2020); Lin et al. (2023); Liu et al. (2023); Lu et al. (2022); Ma et al. (2024); Xin et al. (2024); Xu et al. (2022); Yang et al. (2022); Zheng et al. (2022)	1,210	0.23 [0.12, 0.43]	*p* < 0.00001	10%
Hypertension	10 Chen et al. (2023); Cui et al. (2023); Eberl et al. (2020); Lin et al. (2023); Liu et al. (2023); Ma et al. (2024); Wang et al. (2019); Xin et al. (2024); Xu et al. (2022); Zhan et al. (2022)	1,202	1.75 [0.75, 4.06]	*p* = 0.19	56%
Tachycardia	7 Chen et al. (2023); Cui et al. (2023); Eberl et al. (2020); Liu et al. (2023); Ma et al. (2024); Xin et al. (2024); Xu et al. (2022)	866	1.72 [0.76, 3.93]	*p* = 0.20	64%
Arrhythmia	2 Cui et al. (2023); Zhan et al. (2022)	395	0.14 [0.01, 2.67 ]	*p* = 0.19	-
Injection pain	5 Liu et al. (2023); Lu et al. (2022); Xu et al. (2022); Zhan et al. (2022); Zhan et al. (2022)	629	0.37 [0.25, 0.53]	*p* < 0.00001	29%
Cough	4 Feng et al. (2022); Lin et al. (2023); Nie et al. (2023); Zhan et al. (2022)	468	0.66 [0.33, 1.31]	*p* = 0.24	72%
Body movement	5 Chen et al. (2022); Liu et al. (2023); Xin et al. (2024); Zhan et al. (2022); Zheng et al. (2022)	768	0.60 [0.41, 0.88]	*p* = 0.009	81%
Nausea and/or vomiting	17 Chen et al. (2022); Cui et al. (2023); Eberl et al. (2020); Feng et al., 2022; Lin et al. (2023); Liu et al. (2023); Lu et al. (2022); Ma et al. (2024); Nie et al. (2023); Si et al. (2024); Song et al. (2023); Wang et al. (2019); Xu et al. (2022); Yang et al. (2022); Zhan et al. (2022); Zheng et al. (2023)	2,474	0.76 [0.56, 1.02]	*p* = 0.07	0%
Propofol consumption	5 Chen et al. (2023); Feng et al. (2022); Liu et al. (2023); Nie et al. (2023); Zheng et al. (2023)	464	−1.38 [−2.64, −0.11]	*p* = 0.03	97%
Psychiatric symptoms	13 Chen et al. (2022); Cui et al. (2023); Feng et al. (2022); Liu et al. (2023); Nie et al. (2023); Si et al. (2024); Song et al. (2023); Wang et al. (2019); Xin et al. (2024); Xu et al. (2022); Yang et al. (2022); Zhan et al. (2022); Zheng et al. (2022)	2052	3.10 [2.11, 4.54]	*p* < 0.00001	45%
Dizziness	8 Feng et al. (2022); Liu et al. (2023); Nie et al. (2023); Song et al. (2023); Wang et al. (2019); Xin et al. (2024); Zhan et al. (2022); Zheng et al. (2022)	1,466	1.19 [0.83, 1.71]	*p* = 0.34	54%
Awakening time	6 Chen et al. (2023); Feng et al. (2022); Lu et al. (2022); Nie et al. (2023); Xin et al. (2024); Zheng et al. (2022)	644	0.13 [−0.59, 0.85]	*p* = 0.73	94%
Recovery time	4 Chen et al. (2022); Lin et al. (2023); Liu et al. (2023); Xu et al. (2022)	381	−0.11 [−0.58, 0.35]	*p* = 0.63	77%
Orientation recovery time	5 Chen et al. (2022); Lu et al. (2022); Wang et al. (2019); Zhan et al. (2022); Zheng et al. (2022)	680	−0.05 [−0.21, 0.12]	*p* = 0.56	29%
Children					
Oxygen desaturation	5 Wang et al. (2022); Xu et al. (2022); Zheng et al. (2022); Zheng et al. (2022); Zhong et al. (2023)	594	0.60 [0.35, 1.02]	*p* = 0.06	47%
Hypotension	3 Wang et al. (2022); Xu et al. (2022); Zheng et al. (2022)	322	0.59 [0.37, 0.95]	*p* = 0.03	0%
Cough and body movement	3 Pees et al. (2003); Wang et al. (2022); Xu et al. (2022)	306	0.85 [0.41, 1.76]	*p* = 0.66	0%
Nausea and/or vomiting	6 Pees et al. (2003); Wang et al. (2022); Xu et al. (2022); Zheng et al. (2022); Zheng et al. (2022); Zhong et al. (2023)	670	0.64 [0.25, 1.61]	*p* = 0.34	0%
Emergence delirium	3 Xu et al. (2022); Zheng et al. (2022); Zhong et al. (2023)	275	1.63 [0.06, 44.29]	*p* = 0.77	81%
Visual disturbance	3 Wang et al. (2022); Zheng et al. (2022); Zheng et al. (2022)	411	6.62 [2.18, 20.13]	*p* = 0.0009	0%
Dizziness	4 Wang et al. (2022); Zheng et al. (2022); Zheng et al. (2022); Zhong et al. (2023)	483	1.99 [1.17, 3,37]	*p* = 0.01	0%
Awakening time	2 Xu et al. (2022); Zheng et al. (2022)	311	−0.79 [−2.71, 1.14]	*p* = 0.42	98%
Recovery time	2 Wang et al. (2022); Zhong et al. (2023)	191	0.66 [−0.15, 1.47]	*p* = 0.11	84%

Subgroup analysis of esketamine regimens showed a significant reduction in the risk of oxygen desaturation (RR = 0.51, 95%CI = [0.35, 0.74], *p* = 0.0003); hypotension (RR = 0.38, 95%CI = [0.31, 0.46], *p* < 0.00001); and bradycardia (RR = 0.24, 95%CI = [0.12, 0.47], *p* < 0.0001) in the esketamine-propofol combination, and a marginally significant reduction (*oxygen desaturation*: RR = 0.23, 95%CI = [0.05, 1.06], *p* = 0.06, and test for subgroup differences *p* = 0.32; *hypotension*: RR = 0.26, 95%CI = [0.07, 1.00], *p* = 0.05, and test for subgroup differences *p* = 0.59; *bradycardia*: RR = 0.14, 95%CI = [0.02, 1.12], *p* = 0.06, and test for subgroup differences *p* = 0.64) in the esketamine-non-propofol combination in adults. No significant difference was found in nausea and/or vomiting in the subgroup analysis of esketamine regimens (Table 4). Given that only one study involved esketamine dose >0.5 ug/kg (Lu et al., 2022), we did not perform subgroup analysis of the esketamine dose in adults. Additionally, the pooled results of adults were not altered after eliminating this single esketamine>0.5 ug/kg study (data not shown).

**TABLE 4:** Adults: esketamine drug regimens subgroup analysis (combination of esketamine with propofol *versus* other drugs).

Outcome indicators	Studies	RR [95%]	*p*-value	I^2^
Oxygen desaturation				
esketamine + propofol	15 Chen et al. (2022); Chen et al. (2023); Cui et al. (2023); Eberl et al. (2020); Feng et al. (2022); Liu et al. (2023); Ma et al. (2024); Nie et al. (2023); Si et al. (2024); Song et al. (2023); Xin et al. (2024); Xu et al. (2022); Zhan et al. (2022); Zheng et al. (2023)	0.51 [0.35, 0.74]	*p* = 0.0003	57%
esketamine + non-propofol	2 Lin et al. (2023); Lu et al. (2022)	0.23 [0.05, 1.06]	*p* = 0.06	0%
Hypotension				
esketamine + propofol	14 Chen et al. (2022); Chen et al. (2023); Cui et al. (2023); Eberl et al. (2020); Feng et al. (2022); Liu et al. (2023); Ma et al. (2024); Si et al. (2024); Song et al. (2023); Xin et al. (2024); Xu et al. (2022); Yang et al. (2022); Zhan et al. (2022); Zheng (a) et al., 2023	0.38 [0.31, 0.46]	*p* < 0.00001	45%
esketamine + non-propofol	2 Lin et al. (2023); Lu et al. (2022)	0.26 [0.07, 1.00]	*p* = 0.05	0%
Bradycardia				
esketamine + propofol	9 Chen et al. (2023); Cui et al. (2023); Eberl et al. (2020); Liu et al. (2023); Ma et al. (2024); Xin et al. (2024); Xu et al. (2022); Yang et al. (2022); Zheng et al. (2023)	0.24 [0.12, 0.47]	*p* < 0.0001	17%
esketamine + non-propofol	2 Lin et al. (2023); Lu et al. (2022)	0.14 [0.02, 1.12]	*p* = 0.06	-
Nausea and/or vomiting				
esketamine + propofol	15 Chen et al. (2022); Cui et al. (2023); Eberl et al. (2020); Feng et al. (2022); Feng et al. (2022); Liu et al. (2023); Ma et al. (2024); Nie et al. (2023); Si et al. (2024); Song et al. (2023); Wang et al. (2019); Xu et al. (2022); Yang et al. (2022); Zhan et al. (2022); Zheng et al. (2023)	0.79 [0.57, 1.09]	*p* = 0.15	0%
esketamine + non-propofol	2 Lin et al. (2023); Lu et al. (2022)	0.58 [0.25, 1.38]	*p* = 0.22	0%

### 3.3 Pooled results of children

**FIGURE 3:**
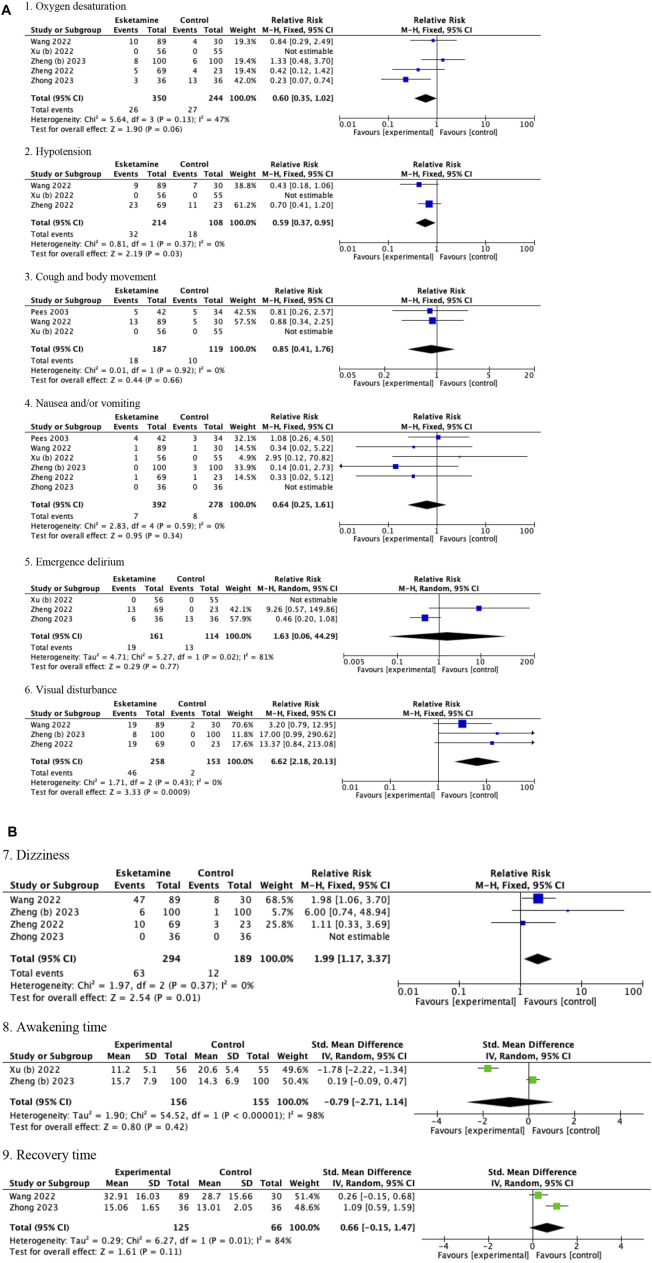
**(A)** Forest plots of children (1. Oxygen desaturation, 2. Hypotension, 3. Cough and body movement, 4. Nausea and/or vomiting, 5. Emergence delirium, and 6. Visual disturbance). **(B)** Forest plots of children (7. Dizziness, 8. Awakening time, and 9. Recovery time).

The pooled results in children showed that compared with drug regimens of the control group, esketamine-propofol combination significantly reduced the risk of hypotension (RR = 0.59, 95%CI = [0.37, 0.95], *p* = 0.03) and increased the risk of visual disturbance (RR = 6.62, 95%CI = [2.18, 20.13], *p* = 0.0009) and dizziness (RR = 1.99, 95%CI = [1.17, 3,37], *p* = 0.01). No significant differences were found in oxygen desaturation (RR = 0.60, 95%CI = [0.35, 1.02], *p* = 0.06); cough and body movement (RR = 0.85, 95%CI = [0.41, 1.76], *p* = 0.66); nausea and/or vomiting (RR = 0.64, 95%CI = [0.25, 1.61], *p* = 0.34); emergence delirium (RR = 1.63, 95%CI = [0.06, 44.29], *p* = 0.77); awakening time (SMD = −0.79, 95%CI = [−2.71, 1.14], *p* = 0.42); and recovery time (SMD = 0.66, 95%CI = [−0.15, 1.47], *p* = 0.11) compared with drug regimens of the control group (Figures 3A,B; Table 3).

Because only one study used the esketamine-midazolam combination, which reported data on nausea and/or vomiting and body movement, we did not conduct a subgroup analysis of the esketamine regimens in children (Pees et al., 2003). After eliminating this single esketamine-midazolam study, the pooled results of children was not changed (data was not shown). Esketamine dose subgroup analysis showed a significant decrease in the risk of hypotension (RR = 0.33, 95%CI = [0.15, 0.75], *p* = 0.008) and dizziness (RR = 2.36, 95%CI = [1.34, 4.18], *p* = 0.003) in the esketamine>0.5 mg/kg subgroup, but a non-significant difference in the esketamine≤0.5 mg/kg subgroup (*hypotension*: RR = 0.73, 95%CI = [0.45, 1.18], *p* = 0.19, and test for subgroup differences *p* = 0.11; *dizziness*: RR = 1.72, 95%CI = [0.98, 3.02], *p* = 0.06, and test for subgroup differences *p* = 0.44). No significant difference was found in oxygen desaturation, nausea and/or vomiting, and visual disturbance in the esketamine dose subgroup analyses (Table 5).

**TABLE 5:** Children: esketamine dose subgroup analysis (≤0.5 mg/kg *versus* >0.5 mg/kg).

Outcome indicators	Studies	RR [95%]	*p*-value	I^2^ ^(%)^
Oxygen desaturation				
esketamine≤0.5 mg/kg	5 Wang et al. (2022); Xu et al. (2022); Zheng et al. (2023); Zheng et al. (2022); Zhong et al. (2023)	0.63 [0.29, 1.37]	*p* = 0.24	44
esketamine>0.5 mg/kg	2 Wang et al. (2022); Zheng et al. (2022)	0.53 [0.17, 1.73]	*p* = 0.30	0
Hypotension				
esketamine≤0.5 mg/kg	3 Wang et al. (2022); Xu et al. (2022); Zheng et al. (2022)	0.73 [0.45, 1.18]	*p* = 0.19	0
esketamine>0.5 mg/kg	2 Wang et al. (2022); Zheng et al. (2022)	0.33 [0.15, 0.75]	*p* = 0.008	12
Visual disturbance				
esketamine≤0.5 mg/kg	3 Wang et al. (2022); Zheng et al. (2022); Zheng et al. (2022)	6.74 [2.19, 20.79]	*p* = 0.0009	0
esketamine>0.5 mg/kg	2 Wang et al. (2022); Zheng et al. (2022)	5.40 [1.46, 19.93]	*p* = 0.01	37
Dizziness				
esketamine≤0.5 mg/kg	4 Wang et al. (2022); Zheng et al. (2022); Zheng et al. (2022); Zhong et al. (2023)	1.72 [0.98, 3.02]	*p* = 0.06	4
esketamine>0.5 mg/kg	2 Wang et al. (2022); Zheng et al. (2022)	2.36 [1.34, 4.18]	*p* = 0.003	0
Nausea and/or vomiting				
esketamine≤0.5 mg/kg	5 Wang et al. (2022); Xu et al. (2022); Zheng et al. (2022); Zheng et al. (2022); Zhong et al. (2023)	0.33 [0.09, 1.24]	*p* = 0.10	0
esketamine>0.5 mg/kg	3 Pees et al. (2003); Wang et al. (2022); Zheng et al. (2022)	1.05 [0.33, 3.30]	*p* = 0.93	0

### 3.4 Sensitivity analysis

We conducted a sensitivity analysis of the primary outcomes (cardiorespiratory adverse events) by eliminating one included study each time. There was no significant difference in the sensitivity analysis in adults. In children, the pooled results of oxygen desaturation were altered after eliminating the study by Zheng (b) 2023 (Zheng (b) et al., 2023), and that of hypotension was altered after eliminating the studies by Wang et al., 2022; Zheng et al. 2022; Wang et al., 2022; Zheng et al., 2022). Due to concerns regarding a high risk of bias, we performed a sensitivity analysis that excluded the study by Pees et al. (Pees et al., 2003). After this exclusion, the pooled results of cough and body movement, and nausea and/or vomiting in children remained consistent.

### 3.5 Publication bias analysis

We assessed publication bias for six indicators, each reported in no less than 10 studies, including oxygen desaturation, hypotension, bradycardia, hypertension, nausea and/or vomiting, and psychiatric symptoms, and the results of this analysis are shown in Supplementary Material S3. However, the symmetry of the four funnel plots for three of these indicators, such as oxygen desaturation, hypotension, hypertension and psychiatric symptoms, was less than ideal, suggesting a possible publication bias.

## 4 Discussion

In this study, we assessed the safety and efficacy of intravenous esketamine as an adjuvant for sedation or analgesia in adults and children undergoing surgical procedures outside the operating room. The unique pharmacodynamics of esketamine (such as dissociative anesthetic property, sympathetic excitatory activity, and local anesthetic action) and the synergistic effect of drug combinations were considered to account for the reduced risk of hypotension, bradycardia, oxygen desaturation, injection pain, and body movement and decreased propofol consumption (Li et al., 2022; Xu et al., 2022; Zhan et al., 2022; Zheng (a) et al., 2023).

Three recent systematic reviews and meta-analyses have investigated the effect of esketamine on sedation or non-intubated anesthesia (Chen H et al., 2023; Huang et al., 2023; Lian et al., 2023). Unlike our review, which aimed to include all combinations of intravenous esketamine, these studies focused specifically on the safety and efficacy of esketamine-propofol combination in comparison to other drug regimens. Our main findings were generally consistent with these three studies, except for one study by Huang et al., which included seven RCTs with 808 patients and reported no significant differences in respiratory depression and body movement with esketamine administration for procedural sedation, without providing a rationale for these finding (Huang et al., 2023). In addition, Chen H et al. included data only from adults, analyzed 14 RCTs and demonstrated that the esketamine-propofol combination provided more stable haemodynamic indices during induction of non-intubated anaesthesia (Chen H et al., 2023). Lian et al., pooling data from 18 RCTs involving 1962 patients, observed that the addition of esketamine to propofol significantly reduced recovery time compared with the saline group, but not compared with the opioid group. They further demonstrated that esketamine significantly lowered the required dose of propofol and the risk of overall complications when compared with both the saline and opioid groups (Lian et al., 2023).

In children, this study first demonstrated that esketamine adjunct to propofol sedation probably caused a lower risk of hypotension but a higher risk of visual disturbance and dizziness. Unlike in adults, the combined use of esketamine and propofol in children provided a limited benefit on the incidence of oxygen desaturation. This finding was consistent with that reported in two systematic reviews with ketamine-propofol combination, indicating only a reduced risk of hypotension and/or bradycardia but not oxygen desaturation in the pediatric population (Foo et al., 2020; Hayes et al., 2021). The possible explanation for these findings is the different respiratory physiology of children such as vulnerable airways, low functional residual capacity of the lungs, poor oxygen reserves, and high oxygen consumption and basal metabolic rate, which makes them more susceptible to respiratory depression from anesthetics (Zheng (b) et al., 2023; Zhong et al., 2023). All respiratory depression events were alleviated by airway management or mask oxygen delivery, and no serious complications occurred in children among the included studies (Wang et al., 2022; Xu (b) et al., 2022; Zheng (b) et al., 2023; Zheng et al., 2022; Zhong et al., 2023).

Another concern about esketamine usage is the psychic emergence reactions that manifest as vivid dreaming; extracorporeal experiences; and illusions with excitement, confusion, euphoria, and fear, and these are often accompanied by auditory and visual disturbances (Miller, 2006; Zhuang et al., 2009). They occur in the first hour of emergence and usually abate within one to several hours (Miller, 2006; Zhuang et al., 2009). Our results showed that the administration of esketamine to adults increased the incidence of psychiatric symptoms. These esketamine-related psychiatric symptoms did not require additional medical support in any of the included studies. Additionally, our study also observed similar awakening, recovery, and orientation recovery times in adults.

It is difficult for young children to express their experience and to self-report potential psychiatric symptoms (Xu (b) et al., 2022). Thus, adverse reactions such as visual disturbance (usually complaints of diplopia), dizziness, and emergence delirium were assessed instead in children. The incidence of visual disturbance and dizziness varied from 8% to 34.78% and 0%–73.3%, respectively, possibly depending on age, esketamine dose, and different surgical procedures among the included studies (Wang et al., 2022; Xu (b) et al., 2022; Zheng (b) et al., 2023; Zheng et al., 2022; Zhong et al., 2023). Furthermore, we found that visual disturbance was associated with esketamine regardless of its doses, while dizziness was associated with esketamine >0.5 mg/kg in this study. Those two postoperative complications were self-limited and did not require intervention among the included studies (Wang et al., 2022; Xu (b) et al., 2022; Zheng (b) et al., 2023; Zheng et al., 2022; Zhong et al., 2023). Although no significant difference was found, the incidence of emergence delirium (assessed using pediatric anesthesia emergence delirium [PAED] scores) in our study should be interpreted with caution. In contrast to previous studies, one meta-analysis that combined one pediatric study and two adults studies observed that esketamine-propofol combination was associated with an increased risk of emergence agitation (not based on PAED scores) (Huang et al., 2023). It is thought that visual disturbance or dizziness may influence the recovery quality of children (Wang et al., 2022; Zhong et al., 2023). However, no significant difference was found in awakening and recovery time in our review.

Combination with propofol is the most common drug regimen of esketamine for sedation or analgesia, and dexmedetomidine and remimazolam were also added to esketamine in adults in two included studies (Lu et al., 2022; Lin et al., 2023). Dexmedetomidine, a highly selective α2-adrenergic agonist, and remimazolam, a short-acting benzodiazepine, both have fewer cardiovascular and respiratory side effects than propofol (Kim and Fechner, 2022; Zhang et al., 2024). Interestingly, a subgroup analysis of esketamine regimens revealed a significant reduction in the risk of oxygen desaturation, hypotension and bradycardia only with the esketamine-propofol combination but not with the esketamine-dexmedetomidine and -remimazolam combinations. Clinical heterogeneity, sample size and limited number of studies may contribute to the discrepancies in the subgroup analysis of esketamine-based regimens. The results of the subgroup analyses should be interpreted with caution. Further research, particularly RCTs with large sample sizes, is warranted to clarify the impact of esketamine in combination with non-propofol agents on the safety and efficacy of sedation and analgesia.

Our study has some limitations: (i) We did not stratify the main analysis by different types of surgical procedures and drug regimens of the control group. This allowed us to broaden the applicability of the findings beyond a specific context. However, these factors may have caused heterogeneity among some analyses. Other factors such as the different definitions of some outcomes and strategies of esketamine and propofol administration may have also contributed to heterogeneity. (ii) Twenty-three of the 25 included studies (92%) were performed in China, which may have led to bias in the results. (iii) Some continuous variables (such as propofol consumption, awakening time, recovery time, and orientation recovery time) of the included studies were reported as medium or with different measurement units, making them unsuitable for direct data merging (Cui et al., 2023; Eberl et al., 2020; Feng (b) et al., 2022; Ma et al., 2024; Si et al., 2024; Song et al., 2023; Wang et al., 2022; Wang et al., 2019; Xu (a) et al., 2022; Xu (b) et al., 2022; Yang et al., 2022; Zhan et al., 2022; Zheng et al., 2022; Zhong et al., 2023). In our study, these data were excluded from the meta-analysis because we failed to obtain raw data from the authors. After qualitative analysis of these data, we found that esketamine groups was associated with lower propofol consumption in all 10 included studies that were ineligible for the meta-analysis (Eberl et al., 2020; Feng (b) et al., 2022; Ma et al., 2024; Song et al., 2023; Wang et al., 2022; Xu (a) et al., 2022; Xu (b) et al., 2022; Zhan et al., 2022; Zheng et al., 2022; Zhong et al., 2023). In addition, three of the seven “inappropriate” studies showed the significantly shorter awakening time and/or recovery time with esketamine administration (Wang et al., 2019; Eberl et al., 2020; Yang et al., 2022; Zheng et al., 2022; Cui et al., 2023; Ma et al., 2024; Si et al., 2024). (iv) Almost 90% of the included studies (22 out of 25) used drug regimens that comprised esketamine and propofol. We could perform the subgroup analysis of esketamine regimens for some outcomes in adults. Future studies are needed to explore the effect of different esketamine combinations on sedation or anesthesia. (v) Publication bias may exist for oxygen desaturation, hypotension, hypertension, and psychiatric symptoms by visual inspection of funnel plots, suggesting the need for cautious interpretation of results.

## 5 Conclusion

This study showed that intravenous esketamine combinations probably reduced the incidence of several cardiorespiratory depression events (hypotension, bradycardia, and oxygen desaturation); injection pain; body movement; and propofol requirement, but increased the risk of psychiatric symptoms in adults who have undergone different surgical procedures outside the operating room. However, subgroup analysis indicated that the incidence of hypotension, bradycardia, and oxygen desaturation in adults was significantly reduced only with the esketamine and propofol combination. In addition, this study also suggested that the combined use of esketamine and propofol may cause a lower risk of hypotension (esketamine>0.5 mg/kg), but a higher risk of visual disturbance and dizziness (esketamine>0.5 mg/kg) in children who have received sedation or analgesia outside the operating room. Although these short-term side effects usually do not require additional intervention, clinicians should be aware of the potential psychiatric symptoms of esketamine. Given the limited number of studies, future research is needed, particularly RCTs with substantial cohorts, to elucidate the effect of esketamine when combined with agents other than propofol on the safety and effectiveness of sedation and analgesia protocols.

## Data Availability

The original contributions presented in the study are included in the article/Supplementary Material, further inquiries can be directed to the corresponding author.
