# Supplementary material for: Intravenous esketamine as an adjuvant for sedation/analgesia outside the operating room: a systematic review and meta-analysis
Source: Front Pharmacol. 2024 Jul 3;15:1287761. doi: 10.3389/fphar.2024.1287761 (PMC11252540; doi:10.3389/fphar.2024.1287761)
Supplement: Supplementary file 1 [file Table1.DOCX]

Supplementary material 1 The search strategies for PubMed, Embase, Cochrane Central Register of Controlled Trials (CENTRAL) Web of Science and Scopus

1.PubMed from database inception to 19th March 2024

| # | Searches | results |
| --- | --- | --- |
| 1 | esketamine[all] OR “s-ketamine”[all] OR “s(+)-ketamine”[all] OR “(s)-ketamine”[all] | 1855 |
| 2 | analgesia[mh] OR analgesics[mh] OR anesthesia[mh] OR anesthetics[mh] OR anesthesia and analgesia[mh] OR conscious sedation[mh] OR deep sedation[mh] OR hypnotics and sedatives[mh] | 534,646 |
| 3 | analgesia[tw] OR analgesic*[tw] OR anesthesia[tw] OR anesthetic*[tw] OR anaesthesia[tw] OR anaesthetic*[tw] OR sedation[tw] OR hypnotic* [tw] OR sedative*[tw] OR analgosedation[tw] OR painless[tw] | 654,739 |
| 4 | #2 OR #3 | 776,502 |
| 5 | #1 AND #4 | 771 |
| 6 | randomized controlled trial [pt] OR controlled clinical trial [pt] OR randomized [tiab] OR randomised [tiab] OR placebo[tiab] OR drug therapy[sh] OR randomly[tiab] OR trial*[tiab] OR group*[tiab] | 7,873,492 |
| 7 | #5 AND #6 | 554 |

2.Embase from 1975 to 19th March 2024

| # | Searches | results |
| --- | --- | --- |
| 1 | 'esketamine'/exp | 1914 |
| 2 | all field (esketamine OR “s-ketamine” OR “s(+)-ketamine” OR “(s)-ketamine”) | 2664 |
| 3 | #1 OR #2 | 2664 |
| 4 | “analgesia”/exp OR “analgesic agent”/exp OR “anesthesia”/exp OR “anesthetic agent”/exp OR “anesthesiological procedure”/exp OR “sedation”/exp OR “hypnotic sedative agent”/exp OR “hypnotic agent”/exp OR “sedative agent”/exp | 2,878,718 |
| 5 | analgesia:ti,ab,kw OR analgesic*:ti,ab,kw OR anesthesia:ti,ab,kw OR anesthetic*:ti,ab,kw OR anaesthesia:ti,ab,kw OR anaesthetic*:ti,ab,kw OR sedation:ti,ab,kw OR hypnotic*:ti,ab,kw OR sedative*:ti,ab,kw OR analgosedation:ti,ab,kw OR painless:ti,ab,kw | 751,327 |
| 6 | #4 OR #5 | 3,039,712 |
| 7 | #3 AND #6 | 2601 |
| 8 | “randomized controlled trial”/exp OR randomization/exp OR “double blind procedure”/exp OR “single blind procedure”/exp OR “clinical trial”/exp OR “placebo”/exp OR “experimental design”/exp OR “crossover procedure”/exp OR “control group”/exp OR “latin square design”/exp OR “comparative study”/exp OR “evaluation”/exp OR “prospective study”/exp | 4,654,531 |
| 9 | random$:ti,ab,kw OR placebo$:ti,ab,kw OR control$:ti,ab,kw OR prospective$:ti,ab,kw OR volunteer$:ti,ab,kw OR (clin$ NEAR/3 trial$):ti,ab,kw OR ((signl$ OR doubl$ OR trebl$ OR tripl$) NEXT/3 (blind$ OR mask$)):ti,ab,kw | 7,014,625 |
| 10 | #7 AND (#8 OR #9) | 1,200 |

3.CENTRAL from 1949 to 19th March 2024

| # | Searches | results |
| --- | --- | --- |
| 1 | All text (esketamine) OR ("s-ketamine") | 1,531 |
| 2 | MeSH descriptor: [analgesia OR analgesics OR anesthesia OR anesthetics OR anesthesia and analgesia OR conscious sedation OR deep sedation OR hypnotics and sedatives] explode all trees | 65,830 |
| 3 | (analgesia):ti,ab,kw OR (analgesic*):ti,ab,kw OR (anesthesia):ti,ab,kw OR (anesthetic*):ti,ab,kw OR (anaesthesia):ti,ab,kw OR (anaesthetic*):ti,ab,kw OR (sedation):ti,ab,kw OR (hypnotic*):ti,ab,kw OR (sedative*):ti,ab,kw OR analgosedation:ti,ab,kw OR painless:ti,ab,kw | 168,688 |
| 4 | #2 OR #3 | 179,079 |
| 5 | #1 AND 4 | 1103 |

4.Web of Science from database inception to 19th March 2024

| # | Searches | results |
| --- | --- | --- |
| 1 | All field (esketamine OR “s-ketamine” OR “s(+)-ketamine” OR “(s)-ketamine”) | 1961 |
| 2 | All field (analgesia OR analgesic* OR anesthesia OR anesthetic* OR anaesthesia OR anaesthetic* OR sedation OR hypnotic* OR sedative*OR analgosedation OR painless) | 632,694 |
| 3 | #1 AND #2 | 829 |

5.Scopus from database inception to 19th March 2024

| # | Searches | results |
| --- | --- | --- |
| 1 | TITLE-ABS-KEY ( esketamine ) OR TITLE-ABS-KEY ( "s-ketamine" ) OR TITLE-ABS-KEY ( "s(+)-ketamine" ) OR TITLE-ABS-KEY ( "(s)-ketamine" ) | 2,370 |
| 2 | TITLE-ABS-KEY ( analgesia ) OR TITLE-ABS-KEY ( analgesic* ) OR TITLE-ABS-KEY ( anesthesia ) OR TITLE-ABS-KEY ( anesthetic* ) OR TITLE-ABS-KEY ( anaesthesia ) OR TITLE-ABS-KEY ( anaesthetic* ) OR TITLE-ABS-KEY ( sedation ) OR TITLE-ABS-KEY ( hypnotic* ) OR TITLE-ABS-KEY ( sedative* ) OR TITLE-ABS-KEY (analgosedation) OR TITLE-ABS-KEY painless | 1,009,892 |
| 3 | #1 AND #2 | 1144 |
