# Supplementary figures and images for: Intravenous esketamine as an adjuvant for sedation/analgesia outside the operating room: a systematic review and meta-analysis
Source: Front Pharmacol. 2024 Jul 3;15:1287761. doi: 10.3389/fphar.2024.1287761 (PMC11252540; doi:10.3389/fphar.2024.1287761)

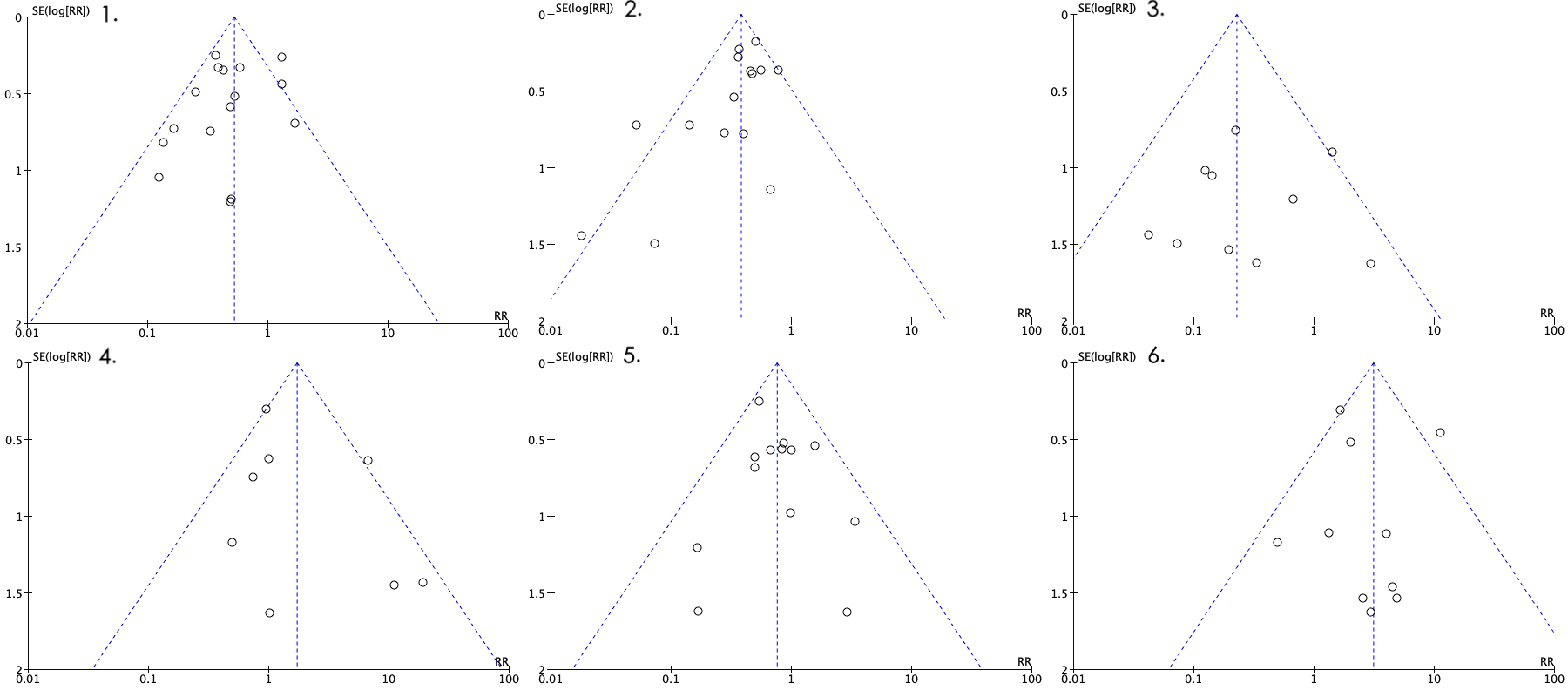

Supplement: Supplementary file 2 [file Image1.JPEG]
