# Supplementary material for: Intravenous esketamine as an adjuvant for sedation/analgesia outside the operating room: a systematic review and meta-analysis
Source: Front Pharmacol. 2024 Jul 3;15:1287761. doi: 10.3389/fphar.2024.1287761 (PMC11252540; doi:10.3389/fphar.2024.1287761)
Supplement: Supplementary file 3 [file Table2.DOCX]

Supplementary material 2 The data of 44 studies excluded by full-text screening

| NO. | Excluded trails | Excluded reasons |
| --- | --- | --- |
| [1] | Esketamine prevents propofol-induced injection pain: Randomized controlled trial. | General anesthesia with intubation |
| [2] | The safety and efficacy of esketamine in comparison to dexmedetomidine during drug-induced sleep endoscopy in children with obstructive sleep apnea hypopnea syndrome: A randomized, controlled and prospective clinical trial. | General anesthesia with intubation |
| [3] | Efficacy and safety of the combination of propofol and S(+)-ketamine for procedural sedation in pediatric patients undergoing totally implantable venous access port implantation: A prospective randomized controlled study. | General anesthesia with intubation |
| [4] | Thermoregulation and haemodynamic during total intravenous anaesthesia with S-(+)-Ketamin/Midazolam, S-(+)-Ketamin/Propofol and Remifentanil/Propofol. | General anesthesia with intubation |
| [5] | Analgesia, sedation and anaesthesia in emergency medicine. | Performed in emergency room |
| [6] | Long-term neurocognitive outcomes after pediatric intensive care: exploring the role of drug exposure. | ICU |
| [7] | Clinical application of esketamine-induced mild sedation technique in outpatient oral surgery. | Oral surgery performed in operating room |
| [8] | Main changes in the 2022 update | Review |
| [9] | Effective dose of propofol combined with a low-dose esketamine for gastroscopy in elderly patients: A dose finding study using dixon’s up-and-down method. | One group |
| [10] | Esketamine as an Adjuvant to Ciprofol or Propofol Sedation for Same-Day Bidirectional Endoscopy: Protocol for a Randomized, Double-Blind, Controlled Trial With Factorial Design. | Protocol |
| [11] | S(+)-ketamine in paediatric anaesthesia. | Editorial |
| [12] | S(+)-Ketamine analgesic drug dose. | Letter |
| [13] | Comparison of the Effects of Esketamine/Propofol and Sufentanil/Propofol on the Incidence of Intraoperative Hypoxemia during Bronchoscopy: Protocol for a Randomized, Prospective, Parallel-Group Trial. | Protocol |
| [14] | What is the best sedation method for high-risk patients such as those with cirrhosis? | Editorial |
| [15] | Low-dose S-ketamine added to propofol anesthesia for magnetic resonance imaging in children is safe and ensures faster recovery - A prospective evaluation. | Letter |
| [16] | Low-dose S-ketamine added to propofol anesthesia for magnetic resonance imaging in children is safe and ensures faster recovery--a prospective evaluation. | Observational cohort study |
| [17] | Effect of inspiratory muscle training on hypoxemia in obese patients undergoing painless gastroscopy: protocol for a single-center, double-blind, randomized controlled trial. | Protocol |
| [18] | Esketamine: Less Drowsiness, More Analgesia. | Review |
| [19] | Influence of ketamine racemate, S(+)-ketamine and R(-)-ketamine in analgesic doses on mental status. | Volunteer study |
| [20] | Low-Dose Esketamine as an Adjuvant to Propofol Sedation for Same-Visit Bidirectional Endoscopy: Protocol for a Multicenter Randomized Controlled Trial. | Protocol |
| [21] | Ketamine racemate or S-(+)-ketamine and midazolam. The effect on vigilance, efficacy and subjective findings | Volunteer study |
| [22] | Erratum: Efficacy and Safety of a Subanesthetic Dose of Esketamine Combined with Propofol in Patients with Obesity Undergoing Painless Gastroscopy: A Prospective, Double-Blind, Randomized Controlled Trial [Corrigendum]. | Erratum |
| [23] | Erratum: efficacy and Safety of a Subanesthetic Dose of Esketamine Combined with Propofol in Patients with Obesity Undergoing Painless Gastroscopy: a Prospective, Double-Blind, Randomized Controlled Trial. | Erratum |
| [24] | Anesthetic management of children undergoing hematologic-oncologic procedures outside the operating room. | Review |
| [25] | Capnographic monitoring of propofol-based sedation during colonoscopy. Endoscopy, 2014, 46(3):236-244. | No outcomes of interest |
| [26] | Median effective dose (ED(50)) of esketamine combined with propofol for children to inhibit response of gastroscope insertion. BMC Anesthesiol, 2023, 23(1):240. | No outcomes of interest |
| [27] | Evaluation of low-dose esketamine on sleep quality in elderly patients undergoing painless gastroscopy. Tropical Journal of Pharmaceutical Research, 2023, 22(4):887-891. | No outcomes of interest |
| [28] | Effect of Esketamine Applied in Fiberoptic Bronchoscopy on Negative Postoperative Behavioral Changes of Children. Iranian Journal of Pediatrics, 2023, 33(6) | Sevoflurane inhalation |
| [29] | Anaesthesia with Midazolam and S (+)-Ketamine in spontaneously breathing patients during magnetic resonance. Anasthesiologie, Intensivmedizin, Notfallmedizin, Schmerztherapie, 1999, 34(Suppl 2):S152. | Rectal administration |
| [30] | Anaesthesia with midazolam and S-(+)-ketamine in spontaneously breathing paediatric patients during magnetic resonance imaging. Paediatr Anaesth, 2000, 10(5):513-519. | Rectal administration |
| [31] | Premedication with orally administered lorazepam in adults undergoing ERCP: a randomized double-blind study. Gastrointest Endosc, 2007, 66(3):450-456. | Group not relevant |
| [32] | Efficacy and safety of Ciprofol for procedural sedation and anesthesia in non-operating room settings. Journal of Clinical Anesthesia, 2023, 85 | Group not relevant |
| [33] | Comparison of remimazolam and propofol combined with low dose esketamine for pediatric same-day painless bidirectional endoscopy: a randomized, controlled clinical trial. Front Pharmacol, 2024, 15:1298409. | remimazolam+ekstamine vs propofol +ekstamine |
| [34] | The Efficacy and Safety of Remimazolam Besylate Combined with Esketamine for Outpatient Colonoscopy: A Prospective, Randomized, Controlled Clinical Trial. Drug Des Devel Ther, 2023, 17:2875-2887. | remimazolam+ekstamine vs proprofol ekstamine |
| [35] | Anaesthesia administered as S(+)-ketamine for cardiac intervention in children with common congenital heart disease. Medicine (Baltimore), 2022, 101(44):e31624. | Grouped by different dose of esketamine |
| [36] | Remimazolam versus propofol in combination with esketamine for surgical abortion: A double-blind randomized controlled trial. Clin Transl Sci, 2023, 16(9):1606-1616. | remimazolam + esketamine vs propofol + esketamine |
| [37] | Effects of opioid-free propofol or remimazolam balanced anesthesia on hypoxemia incidence in patients with obesity during gastrointestinal endoscopy: A prospective, randomized clinical trial. Front Med (Lausanne), 2023, 10:1124743. | remimazolam + esketamine vs propofol + esketamine |
| [38] | Procedural sedation and analgesia for percutaneous trans-hepatic biliary drainage: Randomized clinical trial for comparison of two different concepts. Open Med (Wars), 2020, 15(1):815-821. | remifentanil vs midazolam+ piritramide+ ekstamine + NSAIDs |
| [39] | Efficacy and Safety of a Subanesthetic Dose of Esketamine Combined with Propofol in Patients with Obesity Undergoing Painless Gastroscopy: A Prospective, Double-Blind, Randomized Controlled Trial [Corrigendum] (Drug Des Devel Ther. 2023;17:1347-1356). Drug Design, Development and Therapy, 2023, 17:3231. | Duplicates |
| [40] | Median effective dose (ED<sub>50</sub>) of esketamine combined with propofol for children to inhibit response of gastroscope insertion. Bmc Anesthesiology, 2023, 23(1) | Duplicates |
| [41] | The Clinical Application of Remimazolam Benzenesulfonate Combined with Esketamine Intravenous Anesthesia in Endoscopic Retrograde Cholangiopancreatography. Biomed Res Int, 2022, 2022:5628687. | Retraction |
| [42] | Efficacy of Analgesic Propofol/Esketamine and Propofol/Fentanyl for Painless Induced Abortion: A Randomized Clinical Trial. Biomed Res Int, 2022, 2022:5095282. | Retraction |
| [43] | Effect of Esketamine Added to Propofol Sedation on Desaturation and Hypotension in Bidirectional Endoscopy. Jama Network Open, 2023, 6(12) | Duplicates |
| [44] | ED<sub>50</sub> and ED<sub>95</sub> of propofol combined with different doses of esketamine for children undergoing upper gastrointestinal endoscopy: A prospective dose-finding study using up-and-down sequential allocation method. Journal of Clinical Pharmacy and Therapeutics, 2022, 47(7):1002-1009. | Duplicates |
